# Retraction Note: The fatty liver index (FLI) and incident hypertension: a longitudinal study among Chinese population

**DOI:** 10.1186/s12944-020-01281-z

**Published:** 2020-05-25

**Authors:** Kena Zhou, Jie Cen

**Affiliations:** 1 Ningbo No. 9 Hospital, Ningbo, 315020 China; 2 Ningbo University, Ningbo, 315020 China

**Retraction to: Lipids Health Dis (2018) 17: 214**

**https://doi.org/10.1186/s12944-018-0858-6**

The Editor-in-Chief has retracted this article [1] because it significantly overlaps with a previously published article by Zheng and Mao [2]. The authors have not addressed this, despite several attempts from the Publisher.
